# Elevated temperature, but not decreased pH, impairs reproduction in a temperate fish

**DOI:** 10.1038/s41598-020-77906-1

**Published:** 2020-11-30

**Authors:** Ana F. Lopes, Ana M. Faria, Sam Dupont

**Affiliations:** 1 MARE - Marine and Environmental Sciences Centre, ISPA - Instituto Universitário, 1149-041 Lisbon, Portugal; 2 Department of Biological and Environmental Sciences, University of Gothenburg, 566 Kristineberg, 45178 Fiskebäckskil, Sweden

**Keywords:** Ecology, Environmental sciences

## Abstract

Fish reproductive success is linked to the ability of couples to mate and produce clutches that successfully hatch. Environmental stressors like high temperature and low pH can jeopardize this energetically costly process. In this study, exposure to high temperature and low pH was tested on a marine temperate species, *Gobiusculus flavescens*, to evaluate effects on reproductive performance. Breeding pairs were assigned to different temperatures (+ 0 °C, + 3 °C relative to in situ temperature) and pH levels (8.0, 7.6), in a cross-factorial design for a 3-month period. Reproduction activity, success, and paternal investment were measured throughout the exposure period. Results show reproduction is impaired by elevated temperature, while low pH had little impact. Breeding pairs under high temperature had 3% to 10% hatching success, up to 30% less eggs and eggs up to 20% smaller. Although paternal investment was not affected by tested parameters, males of breeding pairs exposed to elevated temperature had smaller gonadosomatic indexes, which might indicate a lack of investment in the reproductive process. Overall, results show that elevated temperature, expected more frequently in the near future, as a consequence of global warming, may impair key processes like reproduction in temperate fish, with potential consequences for fitness and population replenishment.

## Introduction

Reproduction is an energetically expensive process. Successful reproduction requires investment of time and energy sometimes in detriment of other essential functions that maintain organisms’ fitness^1^. For organisms with multiple reproduction events, sacrificing reproduction over other processes, through prioritization of energy toward vital processes and functions, can increase their chances of survival in a stressing environment. However, disregarding reproduction can put at risk the long-term survival of the species^2^. In the two-spotted goby, *Gobiusculus flavescens*, used in this study, reproduction success includes physiological^3^ and behavioural^4^ processes. Females can allocate energy for partner selection, courtship, egg production, and offspring development^5^, while males energy can be allocated for partner selection, courtship, nest construction and defence^6,7^. The ability of a couple to successfully mate and produce clutches, as well as their potential investment in taking care of the clutches (parental care), is determinant aspects for their offspring’s fitness^4^.

Environmental factors can constraint reproduction of marine organisms^8,9^. Projections for the end of the century suggest an average increase in temperature and *p*CO_2_ in surface ocean up to + 2.5 °C and + 500µatm, respectively^10^. The increase of *p*CO_2_ in the water leads to a process known as ocean acidification, a perturbation of the carbonate chemistry including a decrease in pH. However, the situation is more complex in the coastal zone where natural and anthropogenic events, such as upwelling^11^ and eutrophication^12^, lead to high variability in temperature and *p*CO_2_^13^.

Changes in temperature, one of the most important cues for the onset of the reproductive season, can be responsible for delaying or even interrupting reproductive processes^14^. Fluctuations in the reproductive timings, can in turn, cause a mismatch effect between offspring, food availability and predators^15^. For example, elevated temperatures can disrupt the metabolic activity and foraging behaviours of predators^16,17^ and preys^18^. pH has also been shown to impact reproduction in fish. Some studies showed that reproductive outputs can be up to 50% higher at lower pH, deviating from present environmental pH variability^3,19,20^. Other studies show reduced reproductive output in fish exposed to lower pH, as well as eggs’ abnormalities^20,21^. Impairing reproduction can have severe effects on the offspring quality, however, increasing the investment in reproduction might require using energy that otherwise would be allocated to maintain the homeostasis of the adult organisms^22,23^, and therefore make them more vulnerable to these stressors.

Studies looking at the combined impact of temperature and *p*CO_2_ have showed complex responses depending on the level of exposure^24^, life stages and habitat^25–29^. To date, there are only a few studies on the combined effect of temperature and pH on fish reproductive behaviour and performance. For example, in the tropical fish *Amphiprion melanopus,* clutch survival decreased at higher temperature, with no survival when combined with low pH treatments^30^.

The present study aims at evaluating the effect of temperature and pH on the reproductive performance of a temperate marine fish species, the two-spotted goby, *G. flavescens*. This semi-pelagic species inhabits shallow rocky waters of the European coasts. In Northern Europe, it plays a key ecological role as food of other important commercial fish species, such as *Gadus morhua*^31^. This species has sexual dimorphism and parental care during the embryonic phase, as the males defend the nest and attend to the eggs^6^. The two-spotted goby is also an excellent experimental model. It is easy to maintain and breed in captivity, with minimum stress for the fish^3^.

More specifically, this study evaluates the effect of temperature and pH on (1) the number of clutches laid and successfully hatched; (2) the size and number of eggs per clutch; (3) the larval length at hatch; (4) the parental care behaviour, namely fanning, cleaning, defending and chasing behaviours in the nest; and (5) hepatosomatic and gonadosomatic indexes of the breeding couples, as a proxy for parental investment.

## Results

### Seawater chemistry

A summary of seawater chemistry parameters is presented in Table 1. Temperature and pH were significantly different among treatments (F_1:106_ = 169.98, *p* < 0.001; F_1:106_ = 1412.22, *p* < 0.001, respectively), as expected for the different temperature and pH levels tested. No significant effects were observed for salinity (Supplementary Table S1 online on supplementary materials). As expected, time had a significant effect for temperature and salinity (F_2:106_ = 65.87, *p* < 0.001; F_2:106_ = 9.36, *p* < 0.001, respectively), reflecting seasonal variations in the fjord.

**Table 1:** Seawater chemistry parameters (mean ± s.d.) over the experimental period (June–August) in the 4 treatments: environment (EVM), temperature (T°), pH and temperature*pH (T°xpH). pH on the total scale (pH_T_), temperature (T; °C), salinity (PSU) and total alkalinity (TA; µmol kg^−1^) were used to calculate CO_2_ partial pressure (*p*CO_2_; µatm).

Time	Conditions	Salinity (PSU)	T (°C)	pH_T_	TA (µmol kg^−1)^	*p*CO_2 (µatm)_
June	EVM	24.979 ± 2.648	17.354 ± 1.400	8.062 ± 0.038	2123.084 ± 110.8	397.9 ± 10.558
	T°	24.979 ± 2.648	20.256 ± 0.302	8.024 ± 0.043	2131.17 ± 116.2	448.0 ± 37.447
	pH	24.979 ± 2.648	17.360 ± 1.363	7.614 ± 0.101	2134.997 ± 112.2	1060.7 ± 262.695
	T° × pH	24.979 ± 2.648	20.258 ± 0.281	7.653 ± 0.109	2142.458 ± 113.5	1087.2 ± 256.435
July	EVM	26.685 ± 2.176	19.668 ± 1.443	8.072 ± 0.027	2175.64 ± 44.9	375.4 ± 21.598
	T°	26.685 ± 2.176	22.010 ± 0.704	8.039 ± 0.032	2176.898 ± 46.8	416.1 ± 36.009
	pH	26.685 ± 2.176	19.968 ± 1.326	7.569 ± 0.067	2183.645 + 48.5	1399.6 ± 336.586
	T° × pH	26.685 ± 2.176	22.098 ± 0.728	7.591 ± 0.048	2182.43 ± 48.5	1294.0 ± 102.777
August	EVM	27.267 ± 3.150	20.271 ± 1.594	8.066 ± 0.019	2203.399 ± 137.2	399.0 ± 28.557
	T°	27.267 ± 3.150	22.988 ± 0.282	8.036 ± 0.028	2199.927 ± 143.0	432.7 ± 48.613
	pH	27.267 ± 3.150	20.167 ± 1.703	7.609 ± 0.012	2199.815 ± 158.2	1256.4 ± 74.045
	T° × pH	27.267 ± 3.150	23.129 ± 0.151	7.582 ± 0.012	2196.726 ± 148.2	1353.5 ± 12.263

### Reproductive success

**Figure 1:**
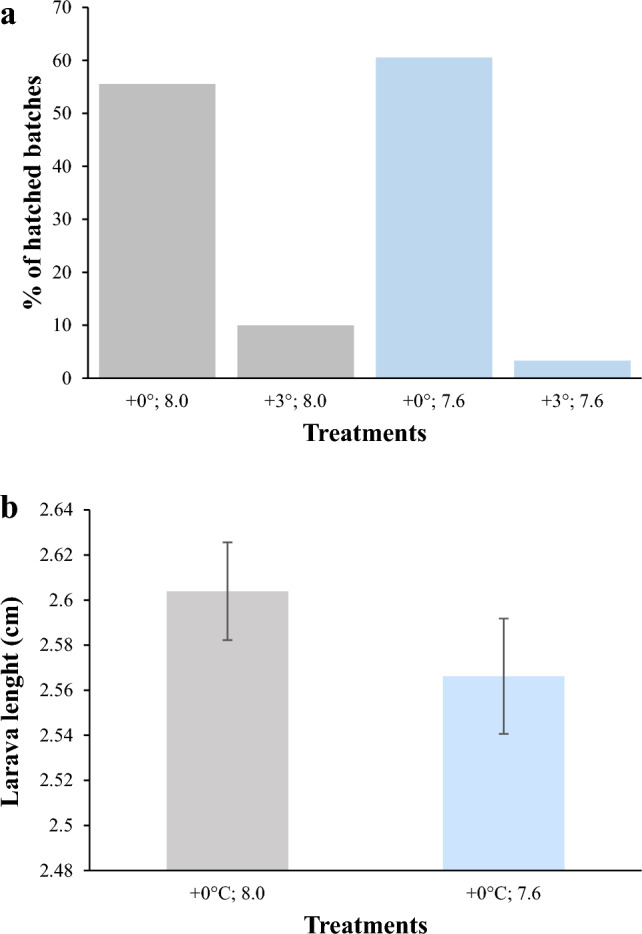
(**a**) Effect of temperature (+ 0 °C, + 3 °C) and pH (8.0, 7.6) on the percentage (%), of hatched clutches. (**b**) Effect of pH (8.0, 7.6) on larva length (cm) at hatching (mean ± S.E.)

Couples breed throughout the entire breeding season. A total of 134 clutches were produced by 29 breeding pairs: 36 laid and 20 hatched in the environment treatment; 30 laid and 3 hatched at high temperature; 38 laid and 24 hatched at low pH; 30 laid and 1 hatched in combined high temperature and low pH. Temperature and pH had no significant impact on the number of clutches laid per breeding pair (Supplementary Table S2 online on supplementary material). The number of clutches that successfully hatched was not influenced by pH (Supplementary Table S2 online on supplementary material). High temperature clutches had 3 to 10% of hatching success (Fig. 1), and this low number of clutches hatched at high temperature prevented a statistical comparison of the hatching success.

**Figure 2:**
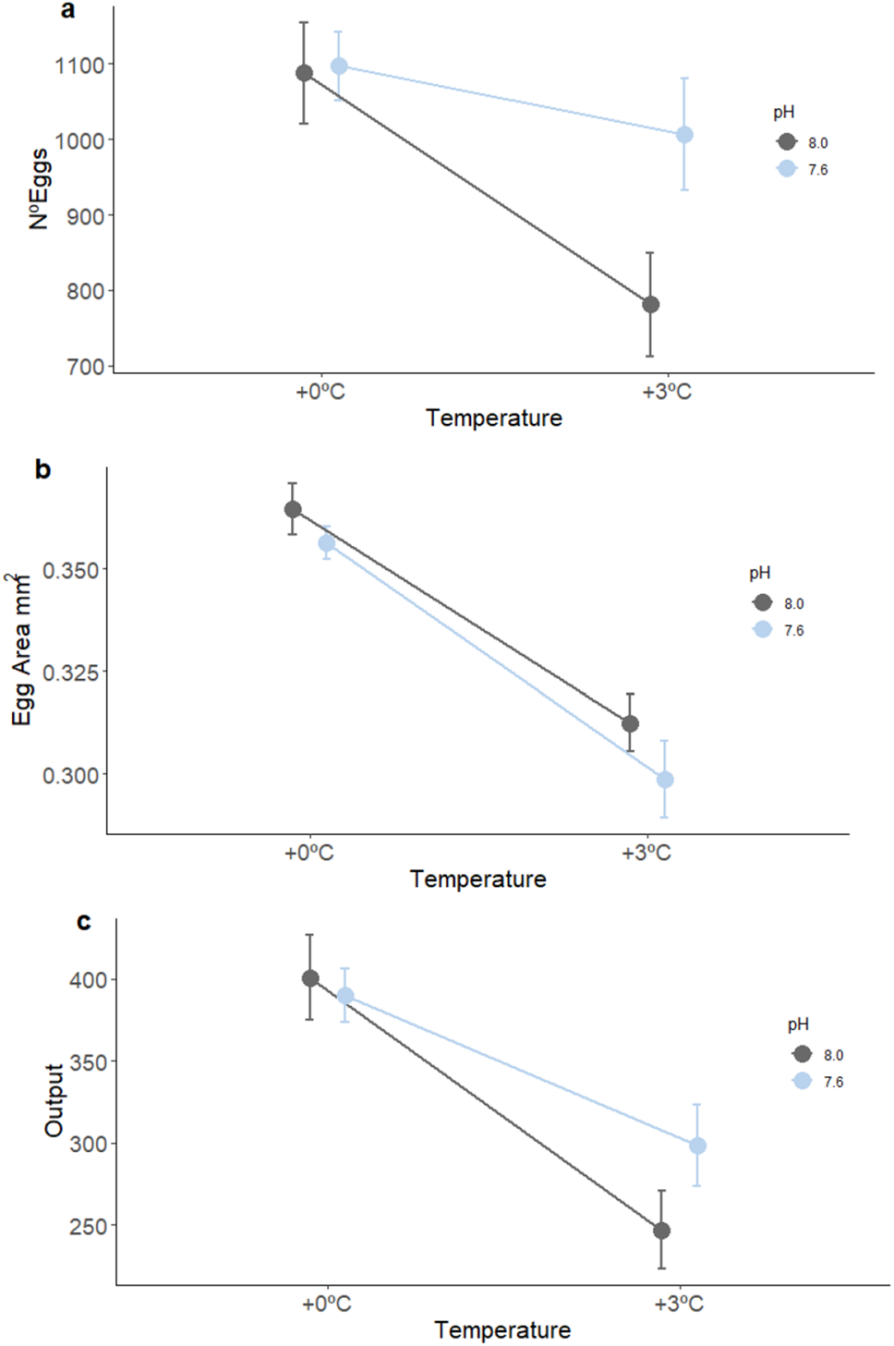
Effect of temperature (+ 0 °C, + 3 °C) and pH (8.0, 7.6) on the number of eggs per clutch (**a**), egg area (**b**), and the reproductive output (**c**) mean ± S.E.

High temperature significantly decreased the average number of eggs per couple (30% less eggs in comparison to the environmental treatment), the egg area (15 to 20% smaller in comparison to the environmental treatment) and the overall reproductive output (number of eggs*mean egg area) (F_1:104_ = 14.24, *p* < 0.001; F_1:104_ = 73.92, *p* < 0.001; F_1:104_ = 41.53, *p* < 0.001, respectively Fig. 2), while low pH and the interaction of temperature and pH had no significant effect (Supplementary Table S2 online on supplementary material). The average number of eggs laid and the overall reproductive output also differed among parental pairs, highlighting intraspecific variability among the couples (Supplementary Table S2 online on supplementary material). The embryonic phase of successfully hatched clutches lasted for 5–6 days, in the environment and pH treatments (Supplementary Table S2 online on supplementary material). pH did not influence the size of the larvae at hatch (Fig. 1), but the length and duration of the embryonic phase differed among parental pairs (Supplementary Table S2 online on supplementary material). The low number of clutches that successfully hatched in the temperature and temperature × pH treatments prevented a statistical comparison of duration of embryonic phase and larval size at hatch under these conditions.

**Figure 3:**
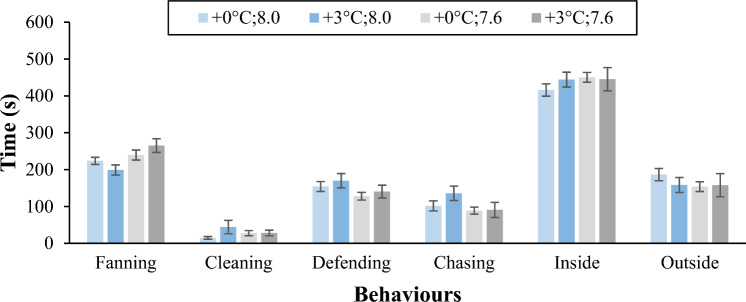
Time (in second; mean ± S.E.) spent by males fanning the eggs; cleaning the eggs; defending the nest; chasing females; and spent inside and outside the nest. All the measures were made from day 2 to day 4 after spawning in the 4 treatments.

Paternal investment, assessed through the measure of time spent on fanning, cleaning, defending and chasing, was not significantly affected by temperature, pH or their interaction (Fig. 3, Supplementary Table S3 online on supplementary material). For all parameters of the behaviour, significant differences among parental pairs were registered (Supplementary Table S3 online on supplementary material), highlighting intraspecific variability among the couples. Males spent more 55% to 65% of the time inside than outside the nest (F_1:169_ = 90,484, *p* < 0.001), as would be expected during the embryonic phase.

No treatment difference was found in males and females across treatments for Fulton’s K (Supplementary Table S4 online on supplementary material). The gonadosomatic index was significantly affected by temperature in males (F_1:24_ = 32.07, *p* < 0.001), but no effect was found in females (Supplementary Table S4 online on supplementary material). No significant results were found in females’ hepatosomatic indexes (Supplementary Table S4 online on supplementary material), while pH had a significant effect in the males’ hepatosomatic index (F_1:24_ = 8.052, *p* = 0.009).

## Discussion

Our results show that reproduction in two-spotted goby is strongly affected by elevated temperature. Couples in all treatments were able to breed, however hatching success was negatively affected by elevated temperature, with only 3–10% of the clutches successful hatched, when compared with the environmental group. High temperature also affected the average number of eggs per couple (less 30% than in environmental couples), and size of the eggs (eggs were 15–20% smaller compared to the environmental treatment).

Similar results were reported in a study where high temperature decreased the hatching success of *Clupea harengus*^32^. Clutches produced under higher temperature had fewer and smaller eggs, and these couples had overall a smaller reproductive output. On the contrary low pH and the interaction of high temperature low pH did not have any effect on the number and size of the eggs. This is consistent with the study on *Amphiprion melanopus*, that found temperature had a greater impact on reproductive performance than CO_2_^30^. Another study on two-spotted gobies, sampled at the same field site, did not find any significant effect of acute exposure to decreased pH (pH 7.6) on spawning or the clutch size^21^. For this population, acclimation times, acute (few days in Forsgren et al.^21^) or chronic (1 month, this study) did not modulate the response to decreased pH. Different results were found for the same species by Faria et al.^3^. Reproduction was stimulated under low pH with an increase in number of clutches and number of eggs. However, this came at a cost, with smaller eggs and larvae at hatch^3^. This is likely a consequence of different experimental conditions, as Faria et al*.*^3^ exposed fish to more extreme *p*CO_2_ levels than the levels in the current study (~ 2300 vs. ~ 1300 µatm, respectively). However, it could also be attributed to a population effect, as Faria et *al.*^3^ tested fish from the Portuguese coast (the southern limit distribution of two-spotted goby), while in the current study fish were sampled from a population that inhabits the Gullmarn fjord, in the Northern Sea. Different populations of a given species can present different sensitivities to pH through local adaptation; populations inhabiting more variable environments being able to tolerate lower pH^33^. The Gullmarn fjord has variable pH with variations up to 0.9 pH units within a single month and pH as low as 7.6 being documented^34^. Our tested scenario (pH 7.6) being within the natural range of variability, it is then not surprising that only mild effects were observed.

We observed a shorter embryonic duration (5–6 days, under all treatments) as compared to the average 10 days of embryonic development reported in other studies^3,21^. This may be a consequence of a particularly warm temperature in Sweden in 2018. The two-spotted goby experiences an average temperature of 16 °C during their reproductive season^21,35^, and in 2018 the maximum recorded temperature during the breeding period reached, 20 °C (Kristineberg Weather Station—https://www.weather.loven.gu.se/kristineberg/en/data.shtml). These record temperatures might have contributed to the observed lower reproductive success under the high temperature treatments.

The two-spotted goby, as most goby species, provide paternal care during the embryonic phase, and, as expected, the time the males spent inside the nest, taking care of the eggs, in all treatments was significantly higher than time spent outside^6,36^. Our results suggest that neither high temperature, low pH or the interaction of both, influences the time males spend on the different parental care behaviours. All these behaviours were recorded from day 2 to day 4 of the embryonic phase. Although males spent time and energy providing care for the eggs, in the end this investment did not pay off in some of the treatments, as the eggs most likely died and were removed by the male. The increase of temperature can increase fungus, infections and general organic matter decomposition can affect the eggs^36^, and would explain why clutches under elevated temperature did not successfully hatch^6^.

Body condition of the two-spotted goby was similar in all treatments, as suggested by the lack of significant differences in Fulton’s K index. A previous study on the effects of low pH on reproductive performance of the two-spotted goby also showed no difference in the K index^3^. Although fish in these treatments can have higher metabolic rates as an exposure to higher temperature, the ad libitum feeding ensured they can compensate for the extra energetically costs and maintain their physical condition under elevated temperature and low pH^29^. The ad libitum food may also have prevented males from eating clutch’s eggs^37^, allowing the focus to be solely on the tested stressors.

GSI was not affected by the treatments for females but was negatively affected by high temperature for males. The gonadosomatic index is used to assess the development of the gonads. Higher values within a reproductive season are usually attributed to the reproduction peak. While low values are attributed to the end of the breeding season^38^. The two-spotted goby have a short life-cycle and reproductive season. Studies on the Gullmarn fjord population mating behaviour showed a transition from strong male–male competition at the beginning of the season, to strong female–female competition later in the season^39^. Our experiment ended in August, close to the end of the breeding season^4^. This can explain the different values of GSI between sexes. Moreover, the significantly lower investment in male gonads at elevated temperature explain the poor quality of the clutches, or why clutches failed to hatch.

pH had a significant effect on males HSI while temperature had no effect. *Acanthochromis polyacanthus* subjected to an increase of 3 °C had larger livers^40^. Larger livers are likely related to higher needs for energy storage under metabolic stress at higher temperature^41^. However, in the two-spotted goby, neither high temperature nor the interaction of high temperature and low pH had an effect on the HSI. Low pH had a significant effect, that might be related to the stress caused by pH. There is no evidence suggesting that liver size directly compromises reproduction, nonetheless indirectly it might influence the size of the gonads (due to space limitation in the abdominal cavity) and overall the reproductive and metabolic processes.

Metabolic demands increase with temperature^32^. This could explain the lower investment in reproductive output at high temperature observed in this study. This is consistent with previous studies showing that temperature influences reproduction in fish^14,42^. Exposure to high temperature within the thermal range fishes are used to endure will make them spend more energy to maintain cellular functions and impair other behaviours not directly essential for their survival^30^. The effect of temperature changes on reproduction is well documented in fish from warmer regions^43^. Studies on species exposed to wider thermal ranges are more scarce. Reproduction performance is an important part of fish development and it is then crucial to understand the impacts of multiple stressors such as pH and temperature in cross-factorial studies^28,44^. Out study shows that exposure to extreme present conditions of temperature and pH already have negative effects on multiple aspects of reproduction. Projections show that such conditions will be more frequent and be more extreme in the near-future. For example, temperature up to 21 °C have been documented in June 2020 in the Gullmarn fjord (Kristineberg Weather Station—https://www.weather.loven.gu.se/kristineberg/en/data.shtml). Without drastic CO_2_ mitigation strategies, local population of two-spotted gobies will then be exposed to more challenging conditions eventually compromising their survival.

Reproduction involves a series of hormonal, physiological and behavioural processes. Future work should consider not only reproductive performance, but also maternal and paternal investment, offspring development, and acclimatization factors in transgenerational experiments. Future works should look into local adaptation in different populations, using the same experimental designs and conditions to test different populations, so results can be compared, as well as other modulating factors, such as food availability. Additionally, transgenerational experiments, where more than one generation is bread in the lab under different environmental conditions, would be important to evaluate adaptation and acclimatization potential to future conditions. Overall, these studies are necessary to better comprehend how reproduction will be affected by global changes, and how will different species, and populations cope with those changes.

## Methods

### Fish collection

The two-spotted gobies were collected in Sweden in the Gullmarsfjord (58° 6 N, 11° 10E), from 25th May to 12th June 2018, by snorkelers using hand-dip nets. In the Gullmarsfjord, the two-spotted goby inhabits seaweed beds on the fjord rocky shores^4^. During breeding season, they seek protected and moderately exposed algal areas in shallow waters^21^.

### Aquarium system

In the laboratory, single sex groups of 8 fish were housed in eight 55L aquaria maintained at 17 °C and pH_T_ 8.00, for a 3-day quarantine period. Flow-through aquaria were used with seawater pumped directly from the fjord at 5 m of depth. After laboratory acclimation, fish were exposed to a total of four treatments: 1) Environment (EVM)- + 0.0 °C × pH_T_ 8.0; 2) pH- + 0.0 °C × pH_T_ 7.6; 3) Temperature (T°)- + 3.0 °C × pH_T_ 8.0; 4) Temperature × pH (T°xpH)- + 3.0 °C × pH_T_ 7.6. pH 7.6 was selected as the average pH in the fjord by 2100 and the extreme of the present natural variability^34^. Eight replicates per treatment were used. Temperature and pH were slowly changed in elevated temperature and low pH aquaria (+ 1 °C/per week; − 0.1 pH/per week), until temperature was + 3 °C as compared to ambient conditions and pH was 7.6. pH was maintained through bubbling of pure CO_2_ and using pH-computers (Aqua Medic, Bissendorf Germany) in each aquarium. In the other aquaria, temperature and pH were maintained at the natural variability of the fjord, throughout the duration of the experiment. High temperature treatments were adjusted throughout the experiment to always maintain a + 3 °C difference from the in situ temperature. Fish were kept under a light cycle of 16/8, and were fed *Artemia* twice a day, ad libitum.

After 1 month of acclimation to the new conditions, all 64 fish were measured and weighed. A male and female were chosen at random from the same treatment, placed in one 30L aquaria, with temperature and pH conditions matching the ones from the acclimation aquaria, and allowed to breed.

### Carbonate chemistry

Temperature and pH (on the total scale—pH_T_) were measured every 2 days. pH probe was calibrated every week using TRIS (Tris/HCL) and AMP (2-aminopyridine/HCl) buffers (salinity 25) following best practices^45^. Total alkalinity (TA) was measured weekly in each aquarium from filtered water samples by titration. Salinity data was obtained from the continuous monitoring of the flow-through system at the Kristineberg station. Other parameters of the carbonate systems parameters were calculated from temperature, salinity, pH_T_ and TA using CO2SYS^46^.

### Endpoints

Breeding couples were provided with a pvc tube (10 cm, Ø3.2 cm) for shelter and nesting. Each pipe was line with a removable acetate sheet, where the spawning females could attach their eggs. Presence of an egg clutch was checked daily. In the presence of eggs, the acetate sheet was carefully removed, placed in a Petri dish filled with seawater from the same aquarium and then photographed for later counting. Approximately 10 eggs were gently removed from the clutch, placed in a smaller Petri dish and photographed under a stereomicroscope for measurement of their surface area. Couples were not affected by the brief removal of the acetate sheet, as the fish resumed normal activity after the acetate sheet was put back in the nest. Males with clutches were recorded with a Panasonic HDC-SD90 camera, for 10 min in the morning 1 h after being fed, as it has been shown that food availability has no effect on parental care for the two spotted goby^6^. Studies showed records of parental care averaging 8 to 20 min are enough to observe fish parental care^36,47^, which corroborates our personal observations. All recordings were done from day 2 to 4 after spawning. Videos were subsequently analysed using the *Observer XT* software (Noldus technology version 5, Netherlands) to quantify the time spent: (1) fanning, (2) cleaning, (3) defending, and (4) chasing the female, as well as (5) total time spent outside and inside the nest. Fanning behaviour consisted of swimming movements with the caudal, dorsal and pelvic fins, to aerate the eggs (adapted from Baklow^48^). Cleaning behaviours included the removal of dead, unfertilized or infected eggs, and debris adapted from Blumer^49^). Defending was defined as the guarding position the male assumed on the nest entry, to avoid intruders. Chasing the female behaviours consisted in charge and chasing the female, outside the nest, to prevent her entrance in the nest.

Reproductive output, as a proxy for maternal investment, was calculated as the overall area (mm^2^) of the clutch by multiplying the total number of eggs per clutch by the average of individual egg area in the same clutch. Close to hatching, the acetate sheet was transferred to a maternity cage (16.5 × 12.5 × 11.5 cm) inside a 55L aquaria matching the temperature and pH conditions of the parental treatment. Ten to 15 larvae were collected at hatching, euthanized using an overdose of MS222, and immediately photographed under a stereomicroscope to measure standard length.

At the end of the experiment, adults were euthanized with an overdose of MS222, weighed and measured, to determine the Fulton’s K condition, K = 100*(W/SL^3^), where W is wet mass in grams and SL is standard length in cm. Fish were immediately dissected and gonads and liver were weighed to determine the gonadosomatic (GSI) and hepatosomatic (HSI) index using the formula: GSI/HSI = (gonad or liver mass (g)/fish mass (g)) * 100.

### Statistical analysis

Data normality was assessed using a Shapiro–Wilk test and homogeneity of variances using a Barlett test. Effect of the different treatments (temperature, pH and time) on the physico-chemical parameters (temperature, pH and salinity) were assessed using a 3 factor linear model Anova (temperature, pH and month). Impact of temperature and pH on the number of clutches laid and hatched, and body condition of the adult fishes (Fulton’s K, gonadosomatic and hepatosomatic indexes) were tested using a 2 factor linear model Anova (temperature and pH). For the number of eggs per clutch, area of the eggs, reproductive output and male reproductive investment, a 3 factor linear model Anova (temperature, pH, and parental pair nested within the treatment) was used. Aquariums with one couple (parental pair) each were replicates within their treatment. Three couples did not reproduce during the experiment, and were excluded from the statistical analysis.

All the statistical analysis was performed with R statistics, RStudio (Version 1.1.463), and results were considered statistically significant at *p* < 0.01.

### Ethics statement

The research was performed in strict accordance with the current Swedish national legislation on Animal Welfare, the experimental procedures were approved by the Swedish Board of Agriculture and the ethical permission was given by the Gothenburg Animal experiment Ethical board under the licence Dnr 103-2014.

## Supplementary information

Supplementary Information 1.
