# Author Correction: Elevated temperature, but not decreased pH, impairs reproduction in a temperate fish

**DOI:** 10.1038/s41598-023-37441-1

**Published:** 2023-06-29

**Authors:** Ana F. Lopes, Ana M. Faria, Sam Dupont

**Affiliations:** 1 MARE - Marine and Environmental Sciences Centre, ISPA - Instituto Universitário, 1149‑041 Lisbon, Portugal; 2 Department of Biological and Environmental Sciences, University of Gothenburg, 566 Kristineberg, 45178 Fiskebäckskil, Sweden

Correction to: *Scientific Reports* 10.1038/s41598-020-77906-1, published online 30 November 2020

The original version of this Article contained errors in the Acknowledgements section.

“Thanks to Fredrik Jutfelt, Roland Pfeiffer, Kirti Ramesh and the staff of the Sven Lovén Center for assistance in the laboratory and field. Thank you also to Rui Oliveira for the use of Observer software, António Roleira for assistance with the software, Sofia Aniceto for the assistance with R software, and Peter Mcgregor on the Advanced Course in Biology and Animal Behaviour (Scientific Writing). This work was supported by the PhD grant of Ana F. Lopes (SFRH/BD/131592/2017), the strategic Project MARE/UIDB/MAR/04292/2020 and the Project NextGen (PTDC/CTA-AMB/31532/2017), co-financed by FCT through national funds and by the European Union through FEDER (European Regional Development Fund). The research leading to these results received partial funding from the European Union’s Horizon 2020 research and innovation programme, grant agreement No 730984, ASSEMBLE Plus Project, and the Clear Reef Social Fund program.”

now reads:

“Thanks to Fredrik Jutfelt, Roland Pfeiffer, Kirti Ramesh and the staff of the Sven Lovén Center for assistance in the laboratory and field. Thank you also to Rui Oliveira for the use of Observer software, António Roleira for assistance with the software, Sofia Aniceto for the assistance with R software, and Peter Mcgregor on the Advanced Course in Biology and Animal Behaviour (Scientific Writing). This work was supported by the PhD grant of Ana F. Lopes (SFRH/BD/131592/2017), the European Regional Development Fund (FEDER) through the Lisbon's Regional Operational Programme (LISBOA-01-0145-FEDER-031532) and the Portuguese Foundation for Science & Technology (FCT) through the project NextGen (PTDC/CTA-AMB/31532/2017) and the strategic project MARE-UIDB/04292/2020 granted to MARE (Marine and Environmental Sciences Centre). The research leading to these results received partial funding from the European Union’s Horizon 2020 research and innovation programme, grant agreement No 730984, ASSEMBLE Plus Project, and the Clear Reef Social Fund program.”

The original Article has been corrected.

