# Supplementary material for: Elevated temperature, but not decreased pH, impairs reproduction in a temperate fish
Source: Sci Rep. 2020 Nov 30;10:20805. doi: 10.1038/s41598-020-77906-1 (PMC7705697; doi:10.1038/s41598-020-77906-1)
Supplement: Supplementary file 1 — Supplementary Information 1. [file 41598_2020_77906_MOESM1_ESM.pdf]

Elevated temperature, but not decreased pH, impairs reproduction in a temperate fish

Ana F. Lopes[a*], Ana M. Faria[a], Sam Dupont[b]

[a] MARE - Marine and Environmental Sciences Centre, ISPA - Instituto Universitário, 1149-041 Lisbon, Portugal.

[b] Department of Biological and Environmental Sciences, University of Gothenburg, 566 Kristineberg, 45178 Fiskebäckskil, Sweden

*Corresponding author: Ana F. Lopes - MARE - Marine and Environmental Sciences Centre, ISPA - Instituto Universitário, 1149-041 Lisbon, Portugal. +351 218811700, email: ana.f.s.lopes@gmail.com

Supplementary Table S1 – Analysis of Variance: effect of temperature (T°), pH, their interaction (T°xpH) and time on parameters of the seawater chemistry (temperature, pH and salinity). Asterisk (*) indicates significant difference (p<0.01).

SOURCES	DF	TEMPERATURE (°C)			pH_T			SALINITY (PSU)		
		MS	F	P	MS	F	P	MS	F	P
**T°**	1	187.100	169.979	<0.001 *	0.0008	0.203	0.653	0.442	0.077	0.783
**pH**	1	0.247	0.225	0.637	5.544	1412.221	<0.001*	0.000	0.0000	0.994
**T° x pH**	1	0.038	0.035	0.853	0.0240	6.080	0.015	0.000	0.0000	0.998
**Time**	2	72.500	65.866	<0.001*	0.0053	1.358	0.261	53.944	9.355	<0.001*

Supplementary Table S2 – Analysis of Variance: A) effect of temperature (T°), pH, their interaction (T°xpH) on number of clutches laid (2 factor Anova) and effect of pH on number of clutches hatched (1 factor Anova); B) effect of temperature (T°), pH, their interaction (T°xpH) and parental pair (PP) nested within temperature*pH, on average number of eggs per clutch, average egg area; reproductive output (3 factor Anova) and on larva length at hatch (2 factor Anova) and duration of embryonic phase. Asterisk (*) indicates significant difference (p<0.01)

**A**		**LAID**			**HATCHED**					
**SOURCES**	**DF**	MS	F	P	MS	F	P			
**T°**	1	10.841	2.263	0.144	-	-	-			
**pH**	1	0.278	0.058	0.811	0.0066	0.0017	0.9679			
**TºxpH**	1	0.298	0.062	0.805	-	-	-			
**B**		**Nº EGGS**			**EGG AREA**			**OUTPUT**		
		MS	F	P	MS	F	P	MS	F	P
**T°**	1	1318463	14.2415	<0.001 *	0.098	73.922	<0.001*	497038	41.525	<0.001*
**pH**	1	367706	3.9718	0.049	0.0037	2.816	0.096	9369	0.783	0.378
**T° x pH**	1	382938	4.1363	0.045	0.0003	0.192	0.662	31550	2.636	0.108
**PP(temp*pH)**	25	298657	3.2260	<0.001*	0.0018	1.373	0.136	38609	3.225	<0.001*
		**LARVA LENGTH**			**EMBRYONIC PHASE**					
**T°**	-	-	-	-	-	-	-			
**pH**	1	0.0138	2.2156	0.1497	0.0495	0.9495	0.3396			
**T° x pH**	-	-	-	-	-	-	-			
**PP(temp*pH)**	13	0.0218	3.5004	0.004*	0.2354	4.519	<0.001*			

Supplementary Table S3 – Analysis of Variance: A) effect of temperature (T°), pH, their interaction (T°xpH) and parental pair(PP) nested within temperature*pH, on time spent by males fanning; cleaning; defending the nest; chasing females; and overall time inside and outside of the nest (3 factor Anova). B) Comparison of time spent outside and inside of the nest in all treatments (1 factor Anova). Asterisk (*) indicates significant difference (p<0.01).

**A**							
**SOURCES**	**DF**	**FANNING**			**CLEANING**		
		MS	F	P	MS	F	P
**T°**	1	2	0.0003	0.985	6939.7	2.9964	<0.086
**pH**	1	33860	5.6698	0.019	1505.3	0.6500	0.421
**T° x pH**	1	20246	3.3902	0.068	6697.9	2.8920	0.091
**PP(temp*pH)**	17	398846	3.9286	<0.001*	4908.7	2.1195	0.009*
		**DEFENDING**			**CHASING**		
**T°**	1	6652	0.919	0.339	10482	1.696	0.195
**pH**	1	31426	4.343	0.039	18324.	2.965	0.087
**T° x pH**	1	87.1	0.012	0.913	8215	1.329	0.251
**PP(temp*pH)**	17	20087	2.776	<0.001*	31505	5.097	<0.001*
		**INSIDE**			**OUTSIDE**		
**T°**	1	4020	0.3564	0.551	4320	0.386	0.535
**pH**	1	29796	2.6419	0.106	26312	2.350	0.127
**T° x pH**	1	8884	0.7877	0.376	8117	0.725	0.400
**PP(temp*pH)**	17	44778	3.9703	<0.001*	2403	3.953	<0.001*
**B**		**Time inside**					
**Time outside**	1	2474572	90484	<0.001*			

Supplementary Table S4 – Analysis of Variance: effect of temperature (T°), pH, their interaction (T°xpH) on body condition of males (A) and females (B): Fulton's K, Gonadosomatic (GSI) and Hepatosomatic (HSI) Indexes (2 factor Anova). Asterisk (*) indicates significant difference (p<0.01).

**A**		**FULTON's K**			**GSI**			**HSI**		
**SOURCES**	**DF**	MS	F	P	MS	F	P	MS	F	P
**T°**	1	0.0204	0.986	0.331	1.984	32.07	<0.001*	0.497	0.768	0.389
**pH**	1	0.051	2.450	0.131	0.0044	0.072	0.791	1.971	8.052	0.009*
**T° x pH**	1	0.0098	0.476	0.497	0.0325	0.526	0.475	0.0059	0.024	0.878
**B**										
**SOURCES**	**DF**	MS	F	P	MS	F	P	MS	F	P
**T°**	1	0.022	1.079	0.309	0.887	0.032	0.860	0.204	0.042	0.840
**pH**	1	0.0098	0.485	0.493	106.18	3.829	0.062	11.89	2.440	0.131
**T° x pH**	1	0.019	0.949	0.340	30.02	1.083	0.308	6.632	1.361	0.255
